# Temporary Inhibition of the Corrosion of AZ31B Magnesium Alloy by Formation of *Bacillus subtilis* Biofilm in Artificial Seawater

**DOI:** 10.3390/ma12030523

**Published:** 2019-02-10

**Authors:** Yaxin Kang, Lei Li, Shunling Li, Xin Zhou, Ke Xia, Chang Liu, Qing Qu

**Affiliations:** 1 School of Chemical Science and Technology, Yunnan University, Kunming 650091, China; 12016000441@ynu.edu.cn (Y.K.); 12015001053@ynu.edu.cn (S.L.); spyxzhou@163.com (X.Z.); 18323999665@163.com (K.X.); cliu6@mail.ynu.edu.cn (C.L.); 2 State Key Laboratory for Conservation and Utilization of Bio-Resources in Yunnan, Yunnan University, Kunming 650091, China

**Keywords:** AZ31B magnesium alloy, biofilm, cyclic voltammetry, corrosion mechanism

## Abstract

It is well known that microorganisms tend to form biofilms on metal surfaces to accelerate/decelerate corrosion and affect their service life. *Bacillus subtilis* was used to produce a dense biofilm on an AZ31B magnesium alloy surface. Corrosion behavior of the alloy with the *B. subtilis* biofilm was evaluated in artificial seawater. The results revealed that the biofilm hampered extracellular electron transfer significantly, which resulted in a decrease of *i*_corr_ and increase of *R_t_* clearly compared to the control group. Moreover, an ennoblement of *E*_corr_ was detected under the condition of *B. subtilis* biofilm covering. Significant reduction of the corrosion was observed by using the cyclic polarization method. All of these prove that the existence of the *B. subtilis* biofilm effectively enhances the anti-corrosion performance of the AZ31B magnesium alloy. This result may enhance the usage of bio-interfaces for temporary corrosion control. In addition, a possible corrosion inhibition mechanism of *B. subtilis* on AZ31B magnesium alloy was proposed.

## 1. Introduction

Microorganisms exhibit a strong affinity to adhere to the surface of metals [1]. This adhesion has the ability to modify the electrochemical environments of the metal/solution interface by forming a biofilm, which leads to the acceleration or deceleration of corrosion reactions [2,3]. Consequently, more and more investigation has been carried out on microbiologically influenced corrosion in recent years [4,5,6]. The corrosion behavior of *Pseudomonas aeruginosa* on Ni-Zn alloy and Ni-Cu alloy was explored by San et al. [7]; they showed that *Pseudomonas aeruginosa* accelerated Ni-Cu alloy corrosion, but inhibited Ni-Co alloy corrosion. Guo et al. [8] proved that the presence of *Bacillus subtilis* enhanced steel’s resistance to corrosion in seawater environment, whereas the existence of *Pseudoalteromonas lipolytica* worsened the alloy’s resistance to corrosion. Based on a dissertation by Duan et al. [9], single species *Desulfovibrio caledoniensis* or *Clostridium* sp. could accelerate the corrosion of carbon steel, but mixed species inhibited it. Batmanghelich et al. [10] discovered that the co-presence of *Desulfovibrio vulgaris* and *Pseudomonas aeruginosa* could inhibit cast iron corrosion more evidently, compared to a single one. As suggested above, the results are not in complete accord when diverse microorganisms or metals are used in various research conditions.

It is worth mentioning that microorganisms can influence corrosion behavior in an advantageous way, the so-called MICI (microbiologically influenced corrosion inhibition) [3,7,11]. Based on the corrosion inhibition ability of bacteria on metals, Örnek et al. [11] proposed a concept called corrosion control using regenerative biofilms. They emphasized that the inhibition ability of corrosion of bacteria should be used to provide a bioremediation method to inhibit localized corrosion of metals. Nevertheless, this research is still in a fundamental stage. This may enlighten the development of a sustainable and biological way for metal corrosion mitigation. For instance, in an oil pipeline environment, the environmental microbial community can be adjusted so that microorganisms with corrosion inhibition function as dominant strains to slow down the corrosion of metal pipelines. Thus, studies of MICI on metals will become progressively more important.

*Bacillus* sp., gram-positive bacteria, aerobic or facultative anaerobic, has strong tolerance to environmental changes [12]. Substantial members of *Bacillus* sp. have been separated out. Among them, *B. subtilis* is widely distributed in aquatic habitats and marine sediments [13,14,15]. Literature reports that the presence of *B. subtilis* could inhibit the localized corrosion of certain metals [16,17,18,19]. The corrosion behavior of *B. subtilis* on cold rolled steel was explored [16], which reported that the latter corrosion rate was significantly reduced in comparison with the initial rate. Örnek et al. [17] observed that the pitting corrosion of Al 2024 was visibly weakened when a *B. subtilis* biofilm was gradually formed. Abdoli et al. [18] showed that the bacteria of *Bacillus* sp. significantly enhanced the anti-corrosion performances of Al coatings. Wadood et al. [19] proved that when injected with *B. subtilis*, the corrosion of 304 Stainless Steel was inhibited. Based on the above literature, it is clear that the anti-corrosion properties of cold-rolled steel, aluminum, and stainless steel have been improved on formation of the *B. subtilis* biofilm. However, the corrosion resistance of *B. subtilis* biofilm to other metals has not been fully explored.

With characteristics such as good biocompatibility, low density and potential antimicrobial activity [20,21,22], magnesium alloys are extensively used in marine industries, biomedical materials, automobile manufacturing, etc. [23,24,25]. The continuous development of the light industry has increased demand for magnesium alloys, especially Mg-Al-Zn [26]. However, magnesium alloys are prone to be eroded [27,28,29,30], limiting practical applications. There are quite a few reasons that can account for the corrosion of magnesium alloys [31,32,33]; for instance, moist or polluted atmosphere, temperature, microorganisms, concentration of Cl^−^, Br^−^, SO_4_^2−^ and NO_3_^−^, pH, etc. Among them, the corrosion effects of microorganisms may play a significant role in analogical problems [34,35,36,37,38]. The corrosion behaviour of sulfate-reducing bacteria (SRB) on Ce-modified cast AZ91 magnesium alloy, AZ91D magnesium alloy, and 2024-T31 Al-Cu-Mg alloy were studied separately [34,35,36], which all indicated that SRB accelerated the corrosion procedure of these alloys. Besides, the corrosion behaviours of AZ31B magnesium alloy caused by the fungi of *Aspergillus niger* and *Trichoderma harzianum* were investigated. The results reflected that both fungi could decrease the resistance of corrosion on the alloy [37,38]. The above corrosion results prove that microbial biofilm can reduce anti-corrosion of magnesium alloy. There is currently no report on the improvement of magnesium alloy’s resistance to corrosion by bacterial biofilm. Therefore, the corrosion behavior of *B. subtilis* biofilm on AZ31B magnesium alloy was systematically researched in this work. 

## 2. Materials and Methods

### 2.1. Bacterium and Medium

*B. subtilis* was supported by the State Key Laboratory for Conservation and Utilization of Bio-resources in Yunnan (Kunming, China). *B. subtilis* was incubated in Luria-Bertani medium (LB). Optical density (OD_550_) of the broth was measured after dilution, with a value of 0.7. Number of colony forming units (CFU) was about 1 × 10^7^ CFU mL^−1^ for the following experiments. 

The study was carried out with artificial seawater medium. The composition is as follows [16], NaCl: 17.6 g L^−1^, MgCl_2_·6H_2_O: 1.87 g L^−1^, Na_2_SO_4_: 1.47 g L^−1^, CaCl_2_·2H_2_O: 0.41 g L^−1^, KCl: 0.25 g L^−1^, NaHCO_3_: 0.08 g L^−1^, KBr: 0.04 g L^−1^, Na_2_HPO_4_: 0.01 g L^−1^, FeSO_4_·7H_2_O: 0.01 g L^−1^, SrCl_2_·6H_2_O: 0.008 g L^−1^, H_3_BO_3_: 0.008 g L^−1^, tryptone: 2.0 g L^−1^, starch: 1.0 g L^−1^, yeast extract: 1.0 g L^−1^, pH: 7.5 ± 0.1. 

### 2.2. Material and Specimen

AZ31B magnesium alloy, 5 mm thick sheets. Each of the alloy coupon was cut to a size of 20 mm × 20 mm. Table 1 displays the chemical compositions of the AZ31B magnesium alloy. 

For the electrochemical measurement, magnesium alloy specimens were made into a working electrode. The alloy samples were connected with copper wire of 20 cm and fixed with epoxy resin glue. The exposed area of the working electrodes in artificial seawater medium was 20 × 20 mm^2^. In the surface topography test, the soaking volume of magnesium alloy samples in artificial seawater medium was 20 × 20 × 5 mm^3^. All the alloy samples were polished sequentially with SiC papers (320–1200 grit), then cleaned with distilled water in an ultrasonic cleaner for 5 min. Under irradiation of an ultraviolet lamp, the samples were disinfected with 75% ethanol and 2.5% glutaraldehyde for 1 h, respectively.

### 2.3. Scanning Electron Microscopy/Energy Dispersive X-ray Spectra Analysis (SEM & EDS)

For SEM & EDS test, the specimens were submerged in 2.5% glutaraldehyde for 15 min, and washed with distilled water. The samples were then dehydrated with different concentrations of alcohol. The type of instrument used for the test is Philips XL30 ESEM-TMP, Amsterdam, Netherlands.

### 2.4. Electrochemical Techniques

#### 2.4.1. Electrochemical Corrosion

To characterize the corrosion behaviour of the samples, electrochemical techniques (cyclic polarization, potentiodynamic polarization, electrochemical impedance spectroscopy (EIS), open circuit potential (OCP)), were carried out by means of an electrochemical workstation from Perkin Elmer Company (PARSTAT 2263, Waltham, MA, USA). A prescriptive three-electrode system was selected, with a platinum plate as the counter electrode, the AZ31B magnesium alloy specimens as the working electrodes, and a saturated calomel electrode (SCE) as the reference electrode.

OCP was conducted for 1000 s with 5 s intervals after stabilization for 10 min at each testing time (12, 24, 48, 96 h); the stable value of *E*_ocp_ at each testing time was then recorded. EIS measurement was performed by a sinusoidal wave with 10 mV amplitude in the frequency range of 10^−1^ to 10^5^ Hz. In the potentiodynamic polarization test, the potential range was −0.25 V to +0.50 V vs. SCE, and scan rate was 0.5 mV s^−1^. Cyclic polarization measurement was conducted with a potential range of −0.50 V to +0.50 V vs. SCE, at a scan rate of 0.5 mV s^−1^. 

#### 2.4.2. Electron Transfer Property 

Electron transfer capacity of *B. subtilis* was measured using a CHI660 electrochemical tester (Chenhua, Shanghai, China). Here, a saturated Ag/AgCl electrode was used as the reference electrode, a platinum wire was used as the counter electrode, and a glassy carbon disk (Ø = 3 mm) was used as the working electrode. The working electrode was polished with alumina powder of 0.3 μm and 0.05 μm. After that, the working electrode was cleaned by ultrasonic with pure water. In the cyclic voltammetry (CV) test, the range of potential was from −0.95 V to +0.10 V vs. Ag/AgCl, and the scan rate was 10 mV s^−1^. In the differential pulse voltammetry (DPV) test, the potential range was from −0.76 V to −0.50 V vs. Ag/AgCl, sampling width was 16.7 ms, potential increment was 4 mV, quiet time was 2 s, pulse width was 50 ms, pulse amplitude was 50 mV, and sensitivity was 10^−6^ A V^−1^.

### 2.5. Ultraviolet-Visible Spectroscopy Analysis (UV-VIS)

In UV-VIS experiments, the corrosion products or *B. subtilis* metabolites were kept separate, and analyzed them by ultraviolet and visible spectroscopy (UV-2401PC, Kyoto, Japan). 

### 2.6. Fourier Transform Infrared Spectroscopy Analysis (FTIR)

For this test, the corrosion products or *B. subtilis* metabolites were separated, and then tested. These samples were carried out by KBr tableting technique. The type of instrument used for the test is Thermo Fisher Scientific Nicolet IS10, Waltham, MA, USA. 

## 3. Results

### 3.1. SEM & EDS

**Figure 1:**
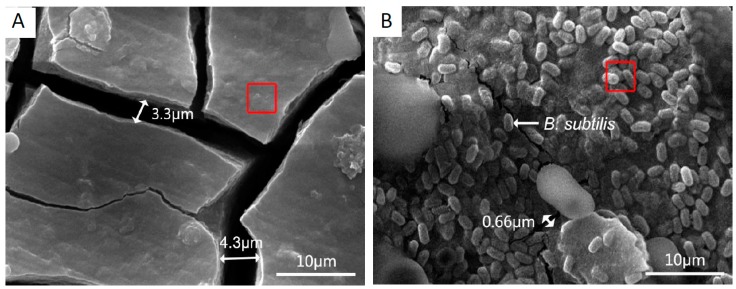
SEM images of AZ31B magnesium alloy specimens in control group (**A**) and *B. subtilis* presence group (**B**) at 48 h. (high voltage: 20 kV).

Figure 1 displays the surface topography of AZ31B magnesium alloy specimens in control group (A) and *B. subtilis* presence group (B) at 48 h. In the control group (Figure 1A), the specimen surface is matt, and mainly has massive and deep large cracks. The largest crack on the specimen’s surface of control group is about 4.30 μm in size. In the *B. subtilis* presence group (Figure 1B), abundant elliptical particles are spread over on AZ31B magnesium alloy surface. In addition, a dense and continuous biofilm of *B. subtilis* was formed on the AZ31B magnesium alloy surface. 

The composition of elements on the surface of specimens is explored by EDS; the test position is the red square in Figure 1A,B. The major elements on the coupons’ surface are presented in Table 2. Compared with the control group, the EDS results of the *B. subtilis* presence group indicate that the content of C, O, P elements has increased. These are key elements of extracellular polymeric substances (EPS) on microbes. EPS consists of proteins, nucleic acids, polysaccharides, lipids, enzymes, etc. [39]. The EDS result demonstrates that a biofilm of *B. subtilis* has been formed on the alloy specimen surface. After electrochemical measurement, the surface corrosion of the alloy is slightly aggravated, but the basic morphology and elemental composition has not changed significantly, as shown in Figure S1 and Table S1 of Supplementary Materials. 

### 3.2. Electrochemical Corrosion

#### 3.2.1. OCP

**Figure 2:**
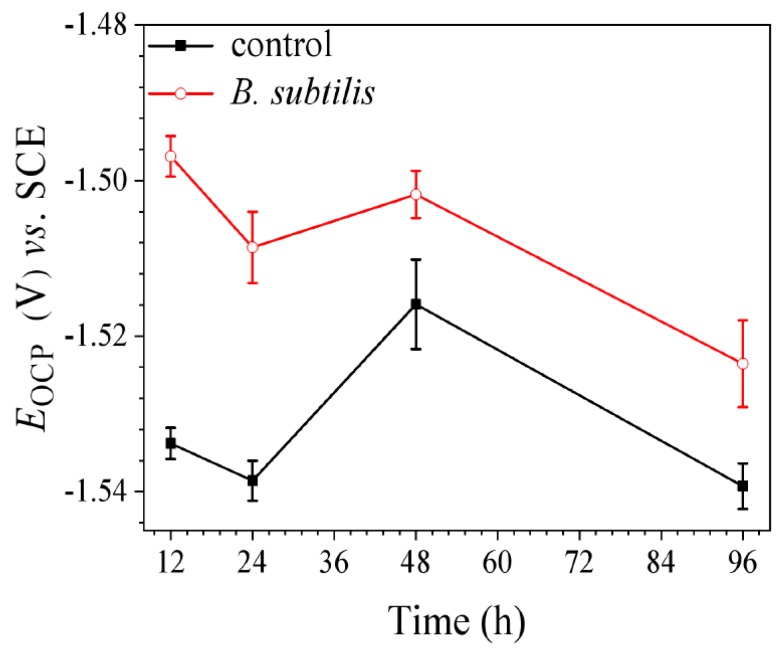
The changes of *E*_ocp_ with immersion time of AZ31B magnesium alloy specimens in control group and *B. subtilis* presence group. Scatter bands represent the standard deviations of the three tests.

The OCP changes with the immersion time of AZ31B magnesium alloy electrodes in the control group and *B. subtilis* presence group (shown in Figure 2). The changing trend of open circuit potential (*E*_ocp_) over time in the *B. subtilis* presence group and control group is in accordance: they all move negatively first, then shift positively, and finally move negatively. However, OCP curves reveal that *E*_ocp_ of the *B. subtilis* presence group is more positive than that of the control group. Some researchers also observed an increase in OCP of different metals with microbes, compared to the sterile ones. Örnek et al. [3,11,17] proposed that an ennoblement of *E*_ocp_ was detected when *B. subtilis* biofilm was present; the biofilm was able to improve the corrosion resistance of aluminum 2024 and brass. Mansfeld [2] pointed out that *Shewanella oneidensis* and *algae* always increased the corrosion rate of metals with a more negative shift of *E*_ocp_, but *B. subtilis* could reduce the corrosion rate with a more positive shift of *E*_ocp_. In these researches, the ennoblement of *E*_ocp_ with *B. subtilis,* compared to the sterile solution, demonstrates that the presence of *B. subtilis* effectively enhances the anti-corrosion performance of AZ31B magnesium alloy.

#### 3.2.2. Potentiodynamic Polarization

**Figure 3:**
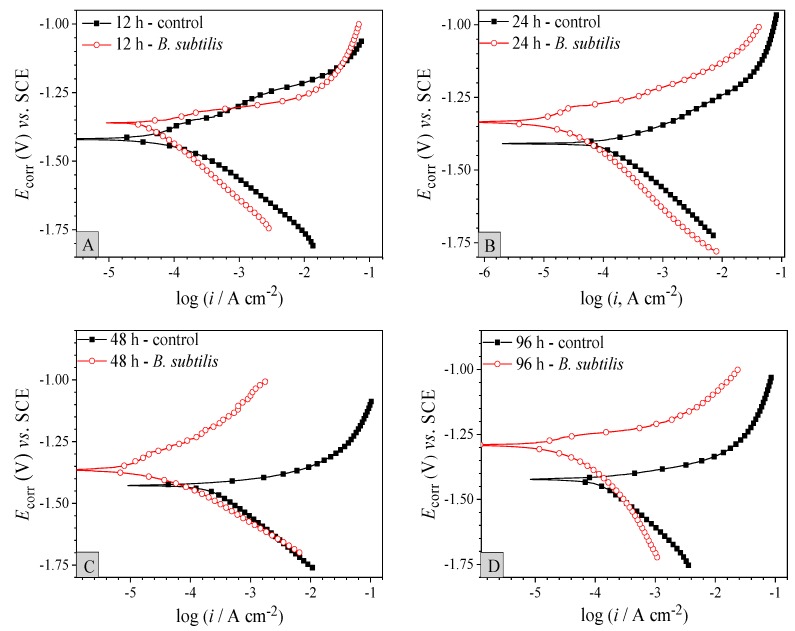
Potentiodynamic polarization curves with immersion time of AZ31B magnesium alloy coupons in the control group and the *B. subtilis* presence group. (**A**) 12 h; (**B**) 24 h; (**C**) 48 h; (**D**) 96 h.

The potentiodynamic polarization variations with immersion time of AZ31B magnesium alloy coupons in control group and *B. subtilis* presence group are shown in Figure 3. The electrochemical parameters are calculated and given in Table 3. Depending on Figure 3, compared with the control group, corrosion potential (*E*_corr_) of the *B. subtilis* presence group increased gradually, and corrosion current density (*i*_corr_) significantly reduced with the prolongation of soaking time, which displays that the presence of *B. subtilis* improves the anti-corrosion performance of AZ31B magnesium alloy. Additionally, Table 3 shows that *i*_corr_ of the control group significantly increased in the early stage, and then slightly decreased. The parameters’ variation of the control group can be because the intrinsic layer (Mg or Al) of the alloy surface is dissolved in the early immersion stage—*i*_corr_ increased progressively. The corrosion products are then amassed gradually on the alloy surface, which form a product film like Mg(OH)_2_, Al(OH)_3_, etc. [40]. The product film hinders contact between the artificial seawater medium and the alloy interface, and causes a slight decline in *i*_corr_. In the *B. subtilis* presence group, *i*_corr_ is clearly decreased and then slightly increased with soaking time. The probable reason is that a compact layer of *B. subtilis* biofilm is formed gradually in the early stage of soaking, which clearly hinders the corrosion of the AZ31B magnesium alloy. Accordingly, *i*_corr_ decreases significantly. However, after 48 h of immersion, the biofilm layer gradually exfoliates due to the consumption of nutrients; *i*_corr_ was found to have increased slightly.

To sum up, *E*_corr_ of the *B. subtilis* presence group increased clearly, and *i*_corr_ reduced significantly, when compared with the control group. This shows that the presence of *B. subtilis* can effectively inhibit corrosion of the AZ31B magnesium alloy.

#### 3.2.3. Cyclic Polarization

**Figure 4:**
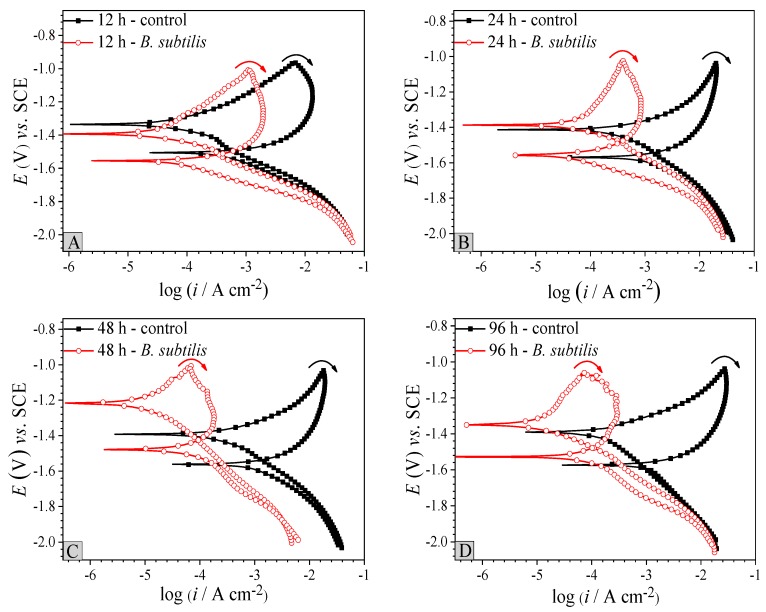
Cyclic polarization curves with immersion time of AZ31B magnesium alloy coupons in the control group and the *B. subtilis* presence group. (**A**) 12 h; (**B**) 24 h; (**C**) 48 h; (**D**) 96 h.

Cyclic polarization is a relatively nondestructive method to evaluate the corrosion susceptibility of materials [41]. Figure 4 displays cyclic polarization variations with the immersion time of AZ31B magnesium alloy coupons in the control group and the *B. subtilis* presence group. Figure 4 shows that all curves appear to be hysteresis loops, protection potential (*E_p_*). Interestingly, the relative area of hysteresis loop in the *B. subtilis* presence group reduced significantly, when compared to the control group. The *E_p_* in the *B. subtilis* presence group is steadily higher than that in the control group. The *E_p_* values of the *B. subtilis* presence group: −1524.3 mV (12 h), −1481.2 mV (24 h), −1431.1 mV (48 h), −1479.4 mV (6 h); the *E_p_* values of the control group: −1492.2 mV (12 h), −543.6 mV (24 h), −1535.0 mV (48 h), −1565.9 mV (96 h). These suggest that corrosion of the AZ31B magnesium alloy is effectively inhibited when it is exposed to the *B. subtilis* environment [41]. In the *B. subtilis* group, *E_p_* reaches a maximum at 48 h, which may be caused by the formation of a compact *B. subtilis* biofilm on the AZ31B magnesium alloy surface. This biofilm hinders contact between the artificial seawater and the alloy surface. The cyclic polarization curves prove that the existence of *B. subtilis* biofilm enhances the anti-corrosion performance of the AZ31B magnesium alloy.

#### 3.2.4. EIS

**Figure 5:**
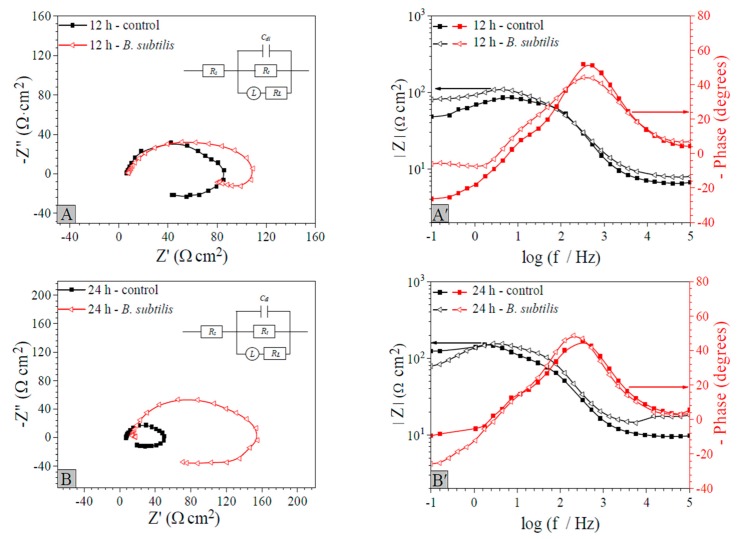

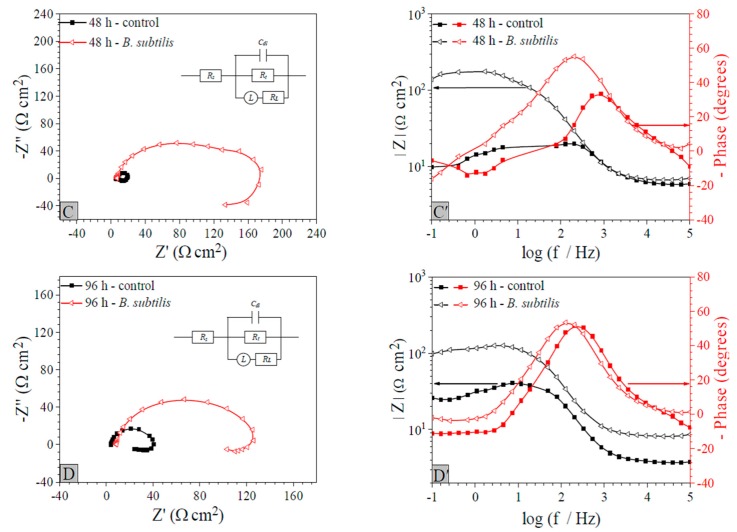
EIS with immersion time of AZ31B magnesium alloy coupons in the control group and the *B. subtilis* presence group. (**A**–**D**): Nyquist diagrams, (**A’**–**D’**): the Bode modulus diagrams and Bode phase angle diagrams.

The corrosion characteristics of AZ31B magnesium samples were performed by EIS after exposure to artificial seawater or inoculation with and without *B. subtilis* (plotted in Figure 5). Figure 5A–D is Nyquist diagrams, Figure 5A’–D’ is Bode modulus diagrams and Bode phase angle diagrams, respectively. All the Nyquist diagrams in Figure 5A–D show analogous and incomplete semicircles. The magnitude of the semicircle diameter reflects the degree of electron transfer. If the diameter is large, it means the electron transfer process is greatly hindered; the electron transfer rate is slow, and displays a small amount of electronic exchange in unit time. In other words, the coupon is corroded difficultly, which appears as corrosion inhibition [42,43,44,45]. Figure 5A–D reflect that the anti-corrosion performance of AZ31B magnesium alloy samples in the *B. subtilis* presence group is increased significantly with immersion time, when compared to the control group, which presumably is attributed to the formation of the *B. subtilis* biofilm. That is, *B. subtilis* biofilm can slightly improve the anti-corrosion characteristics of AZ31B magnesium alloy. An inductive impedance arc appears in a low frequency range in the *B. subtilis* presence group, especially after immersion for 48 h, which is put down to the formation of corrosion source on the AZ31B magnesium alloy surface in the corrosion induction period. In this period, the thickness of *B. subtilis* biofilm is a stated variable; the Faraday current decreased with an increase in thickness of the *B. subtilis* biofilm [46]. Figure 5A–D shows that corrosion resistance is the strongest at 48 h; hence, the thickness of the *B. subtilis* biofilm is the highest at that time. In the control group, the impedance value is decreased significantly and then slightly increased. It is mainly because the magnesium alloy is directly exposed in the artificial seawater medium, the surface is dissolved, and the corrosion rate is gradually increased. However, with time, a corrosion product is gradually formed and covers the alloy surface, which restricts the rapid transmission of erosive ions and molecules to the matrix materials [40]. This leads to an increase of the impedance value of the latter. In the *B. subtilis* presence group, the impedance value is increased firstly and then decreased. It is caused mainly by the formation of the *B. subtilis* biofilm on the AZ31B magnesium alloy surface with immersion time, leading to a clear reduction in corrosion rate. However, after soaking for 48 h, the biofilm layer is gradually exfoliated and the nourishing substances are depleted. Therefore, the impedance value decreases.

The corresponding numerical values of the electrochemical parameters modeled by the equivalent electrical circuit are shown in Table 4. The corrosion inhibition efficiency (*η*) can be obtained by the formula *η* = (*R_t_’ − R_t_*)*/R_t_’ ×* 100%. It can be calculated as: *η* is 14.7% (12 h), 68.8% (24 h), 91.4% (48 h), and 69.4% (96 h), respectively. These prove that the *B. subtilis* biofilm can effectively increase the anti-corrosion performance of the AZ31B magnesium alloy. Moreover, at 48 h, corrosion inhibition is the strongest.

### 3.3. Electron Transfer Property

**Figure 6:**
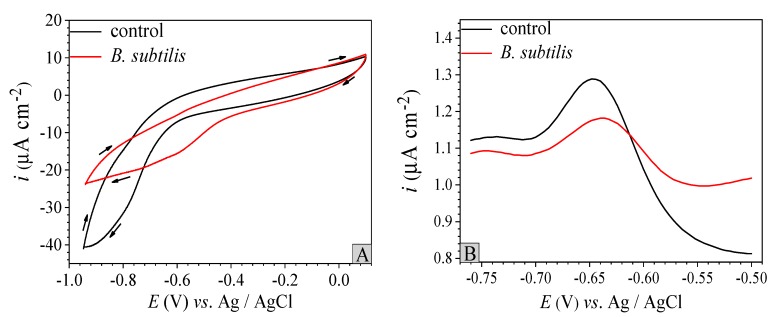
CV curves (**A**) of the control group and *B. subtilis* presence group at 48 h, DPV curves (**B**) of the control group and *B. subtilis* presence group at 48 h.

CV and DPV measurements are shown in Figure 6. Figure 6A presents two CV curves. In the control group, the CV curve shows a weak oxidation peak in the range of −0.7 V to −0.6 V. However, the oxidation peak of the *B. subtilis* presence group is slight. It is almost invisible, when compared to the control group, which suggests that the electronic transmission capacity is weakened by *B. subtilis*. Figure 6B displays two DPV curves. The oxidation peak of the *B. subtilis* presence group is at −0.635 V, the oxidation peak of control group is at −0.645 V, and the current signals in the absence and presence of *B. subtilis* are 12.9 µA cm^−2^ and 11.8 µA cm^−2^, respectively. That is, the current signal of the *B. subtilis* presence group decreases, compared to the control group, which suggests that the *B. subtilis* biofilm inhibits electronic transmission from the electron donor to the electron receptor [47,48,49]. The causation of alterations of CV and DPV in the *B. subtilis* presence group is ascribed to the adhesion of *B. subtilis* cells and EPS on the working electrode surface. These tangibly demonstrate that *B. subtilis* plays an inhibitory role in the electron transfer process.

### 3.4. UV-VIS

**Figure 7:**
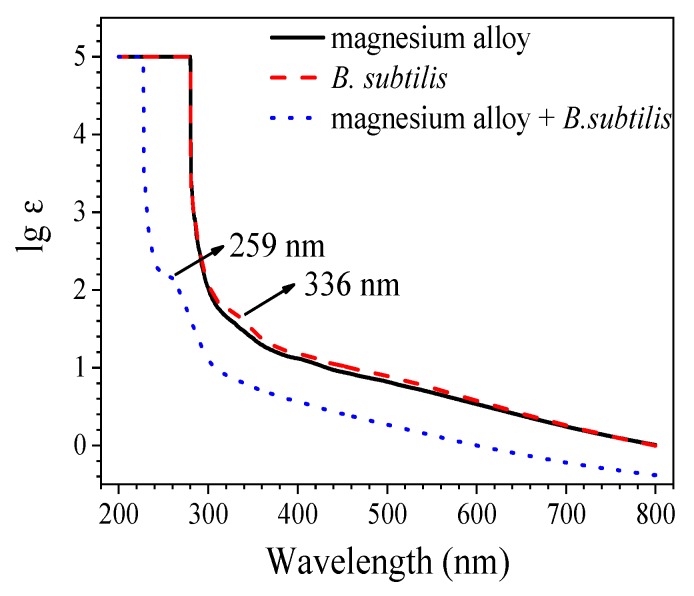
UV-VIS analysis of the AZ31B magnesium alloy, *B. subtilis*, and AZ31B magnesium alloy with inoculation of *B. subtilis* at 48 h.

Figure 7 shows the UV-VIS spectra of *B. subtilis*, AZ31B magnesium alloy inoculation with or without *B. subtilis* after immersion in artificial seawater for 48 h. In the spectrogram of *B. subtilis*, there is a weak absorption peak at 336 nm, which may be caused by the n-π* transition of aldehydes or ketones in the metabolites of *B. subtilis*. The spectrogram of the AZ31B magnesium alloy in the presence of *B. subtilis* is clearly different in the spectrograms of *B. subtilis* and AZ31B magnesium alloy. It has a relatively conspicuous absorption peak at 259 nm, compared with other groups. The absorption peak may be generated by the π-π* transition; it can be supposed that the absorption peak is caused by the interaction between the metabolites of *B. subtilis* and the AZ31B magnesium alloy.

### 3.5. FTIR

**Figure 8:**
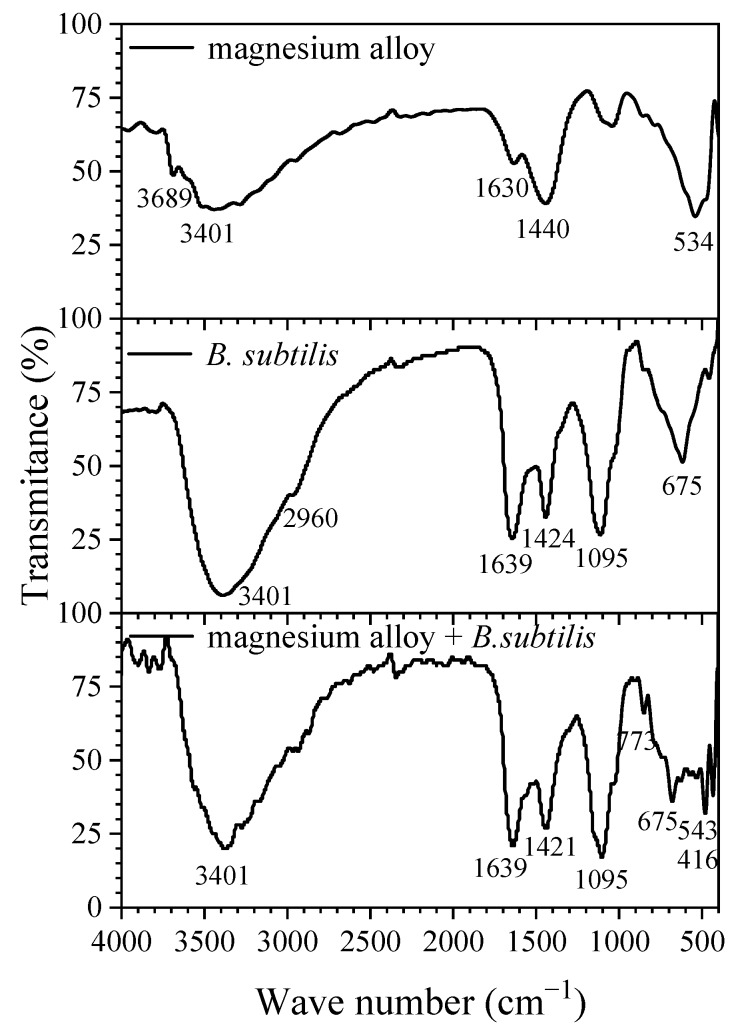
FTIR spectra of AZ31B magnesium alloy, *B. subtilis*, and AZ31B magnesium alloy with inoculation of *B. subtilis* at 48 h.

Figure 8 shows the spectrum graph of the AZ31B magnesium alloy; the absorption peaks of 3689 cm^−1^, 3401 cm^−1^, 1440 cm^−1^ represent an O–H bond and the sharp peak at 534 cm^−1^ is assigned to the Mg–O bond [50]. In the spectrum graph of *B. subtilis*, the peak of 3200–3500 cm^−1^, 1639 cm^−1^, 1095 cm^−1^, 675 cm^−1^ represent the O–H, C–O, C=O bonds of polysaccharides in the metabolic products of *B. subtilis* [51,52,53,54,55,56]. The peaks at 3200–3500 cm^−1^, 1424 cm^−1^ represent N–H, C–N bonds from proteins in the metabolic products of *B. subtilis* [53,54]. When comparing the spectrum graph of the AZ31B magnesium alloy in the presence of *B. subtilis* with the other two graphs, the main difference is that the characteristic peaks at 773 cm^−1^ and 534 cm^−1^, 416 cm^−1^ are caused by the Al–O, Mg–O bond from complexes of metal ion-organic ligand [57]. This means that a dense *B. subtilis* biofilm is formed on the alloy surface, which consists of protein, polysaccharides, etc. Sunde et al. [58] reported that macromolecular hydroxyl compounds have a certain affinity with metal cations and form metal-organic ligand compounds, which facilitate the strong binding of EPS and AZ31B magnesium alloy.

## 4. Discussion

According to the SEM & EDS tests (Figure 1), potentiodynamic polarization (Figure 3), cyclic polarization (Figure 4) and EIS (Figure 5), significant corrosion inhibition occurs when AZ31B magnesium alloy is in artificial seawater medium with a *B. subtilis* biofilm. Outcome of the electron transfer property (Figure 6) indicates that the presence of *B. subtilis* hinders the transfer of electrons. In addition, the investigations of UV-VIS (Figure 7) and FTIR (Figure 8) prove that metal ion-organic ligand complexes are formed on the alloy surface. These complexes can firmly bind EPS to the AZ31B magnesium alloy. From the experiments, we put forward a possible mechanism for the presence and absence of *B. subtilis*.

In the control group, when the alloy is immersed in artificial seawater solution, the surface metals of Mg and Al are dissolved to form corrosion products. These metals in active sites are relatively easier to dissolve, so corrosion occurs through cracks. Anodic dissolution of the magnesium alloy is balanced by the cathodic reduction reaction:

Mg → Mg^2+^ + 2 e^−^ (1)

Al → Al^3+^ + 3 e^−^ (2)

H_2_O + 1/2 O_2_ + 2e^−^ → 2 OH^−^ (3)

**Figure 9:**
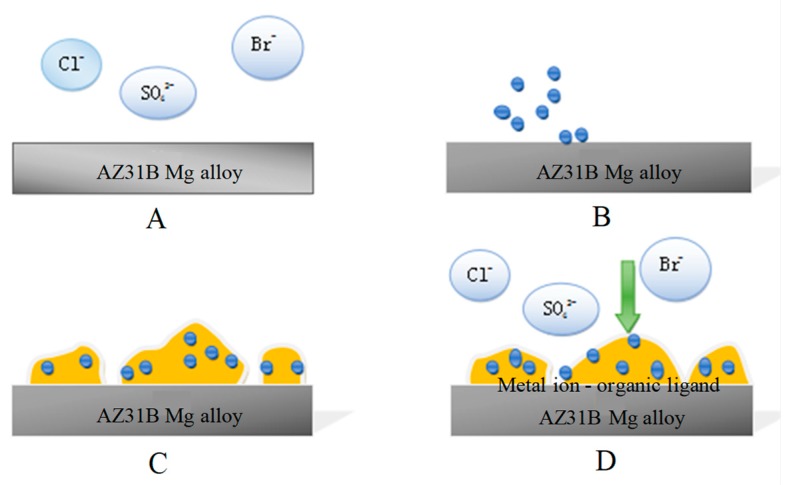
Corrosion effect of *B. subtilis* biofilm on the AZ31B magnesium alloy surface. (**A**) Substrate material; (**B**) Cells adhesion; (**C**) Thin biofilm; (**D**) Dense biofilm.

In the *B. subtilis* presence group, *i*_corr_ exhibits significant decrease compared to the control group. This indicates that the corrosion resistance of AZ31B magnesium alloy is improved. Örnek et al. [17] observed when *B. subtilis* secreted polyaspartic acid or polyglutamic acid, it inhibited corrosion. However, components and concentrations of microbial secretions are affected by mediums with different nutrients. Thus, this mechanism type of inhibition-secretion is not suitable for this study. Based on a review by Xu et al. [1], corrosion mechanism could be divided into aerobic corrosion and anaerobic corrosion. When *B. subtilis* adheres firmly to the surface of the AZ31B magnesium alloy to form a dense biofilm, it is mainly anaerobic corrosion. This dense biofilm hinders the transfer of electrons from the anode to the cathode, which makes corrosion reaction long and tedious, leading to gradual corrosion inhibition of the magnesium alloy [1]. Liu et al. [8] proved that when compared to loose biofilm, the existence of dense and compact *B. subtilis* biofilm enhanced metallic resistance to corrosion. Therefore, an anti-corrosion mechanism of *B. subtilis* biofilm on the alloy surface is shown in Figure 9. In the early stages of immersion, microbes of *B. subtilis* are at a reproductive stage and the alloy surface does not have a biofilm covering. The planktonics of *B. subtilis* cells are preferred for adherence to the alloy surface through electrostatic attraction force (Figure 9B) [59]. Moreover, *B. subtilis* cells metabolize and propagate on the alloy surface, and form a thin biofilm (Figure 9C) [60]. The metabolites of *B. subtilis* then combine with magnesium ions of the alloy surface to form metal ion-organic ligand complexes, which make the biofilm denser and firmer (Figure 9D). The dense biofilm of *B. subtilis* does not only delay the dissolution of the magnesium alloy, but also hinders electron transfer and diffusion, which results in a decrease of *i*_corr_. The anti-corrosion performance of the alloy is thus enhanced. 

## 5. Conclusions

Electrochemical techniques and surface topography analysis methods were used to analyze the corrosion inhibition behaviour of *B. subtilis* biofilm on a AZ31B magnesium alloy surface in artificial seawater. The following conclusions were obtained:

1. *B. subtilis* can form a compact biofilm on the AZ31B magnesium alloy surface.

2. Potentiodynamic polarization, EIS, cyclic polarization, and SEM results significantly inhibit corrosion when a *B. subtilis* biofilm is present on the AZ31B magnesium alloy surface. 

3. The investigation results of UV-VIS, FTIR, CV and DPV testify that complexes such as Al^3+^-organic acid and Mg^2+^-organic acid are formed on the alloy surface. These complexes firmly attach the biofilm to the alloy surface. The biofilm effectively hinders the transfer of electrons and safeguards the alloy.

## Figures and Tables

**Table 1:** Chemical compositions of AZ31B magnesium alloy (wt %).

Element	Al	Zn	Mn	Si	Ni	Fe	Cu	Mg
wt %	3.05	0.99	0.28	0.025	0.0049	0.003	0.002	balance

**Table 2:** Elemental compositions (wt %) of the surface of AZ31B magnesium alloy specimens in the control group and *B. subtilis* presence group.

Element (wt %)	C	O	Na	Mg	Al	P	S	Cl	Ca	Others
The sterile control group	0.81	23.63	0.55	62.52	3.48	0.67	0.37	0.32	7.65	<0.001
The *B. subtilis* presence group	6.11	33.39	2.35	31.61	4.06	11.90	0.57	2.40	7.61	<0.001

**Table 3:** Potentiodynamic polarization parameters with immersion time of AZ31B magnesium alloy coupons in the control group and the *B. subtilis* presence group.

Groups	Time (h)	*E*_corr_ (mV) vs. SCE	*i*_corr_ (μA cm^−2^)	*β_a_* (mV deg^−1^)	*β_c_* (mV deg^−1^)
The sterile control group	12	−1415.4 ± 0.1	83.0 ± 0.5	152.5 ± 9.2	−113.0 ± 3.6
	24	−1410.7 ± 0.1	166.4 ± 0.7	193.2 ± 6.8	−89.8 ± 7.4
	48	−1427.7 ± 0.1	243.3 ± 1.2	203.7 ± 10.5	−52.1 ± 10.2
	96	−1423.1 ± 0.2	116.4 ± 0.8	199.7 ± 11.6	−37.1 ± 6.3
The *B. subtilis* presence group	12	−1362.4 ± 0.1	47.4 ± 0.6	209.7 ± 7.2	−48.1 ± 5.4
	24	−1341.1 ± 0.2	18.3 ± 1.0	189.5 ± 9.3	−52.3 ± 6.8
	48	−1356.9 ± 0.1	14.5 ± 0.9	117.2 ± 9.8	−151.3 ± 11.3
	96	−1291.3 ± 0.1	16.3 ± 0.9	113.1 ± 10.2	−72.1 ± 8.9

**Table 4:** EIS parameters with immersion time of AZ31B magnesium alloy coupons in the control group and the B. subtilis presence group. *R_s_* represents the solution resistance, *C_dl_* represents double layer capacitance, *R_t_* represents charge-transfer resistance, *L* represents equivalent inductance, *R_L_* represents equivalent resistance.

Groups	Time (h)	*R_s_* (Ω cm^2^)	*C_dl_* (×10^−5^ F cm^−2^)	*R_t_* (Ω cm^2^)	*L* (H cm^2^)	*R_L_* (Ω cm^2^)
The sterile control group	12	6.5 ± 0.1	4.1 ± 0.1	76.9 ± 1.8	39.1 ± 1.7	53.2 ± 0.2
	24	7.0 ± 0.2	8.5 ± 0.3	40.9 ± 0.8	18.4 ± 0.9	15.2 ± 0.2
	48	5.9 ± 0.1	2.2 ± 0.3	13.8 ± 2.2	1.8 ± 2.5	8.1 ± 0.23
	96	3.8 ± 0.3	9.3 ± 0.3	36.8 ± 0.5	9.23 ± 0.7	47.4 ± 0.1
The *B. subtilis* presence group	12	7.7 ± 0.2	7.7 ± 0.2	90.2 ± 0.7	40.9 ± 0.9	81.7 ± 0.2
	24	16.0 ± 0.2	3.5 ± 0.2	131.1 ± 0.9	91.6 ± 1.2	67.2 ± 0.2
	48	6.5 ± 0.2	9.8 ± 0.5	161.4 ± 1.2	98.2 ± 2.4	132.8 ± 0.3
	96	8.3 ± 0.2	8.1 ± 0.1	120.3 ± 0.3	63.1 ± 0.8	493.3 ± 0.2

## Supplementary Materials

The following are available online at http://www.mdpi.com/1996-1944/12/3/523/s1, Figure S1. SEM images of AZ31B magnesium alloy specimens before and after electrochemical measurement in the control group (before: A, after: B) and the *B. subtilis* presence group (before: C, after: D) at 48 h. (high voltage: 20 kV). Table S1. Elemental compositions (wt %) of the surface of AZ31B magnesium alloy specimens before and after electrochemical measurement in the control group and the *B. subtilis* presence group.

## References

[1] Jia R., Unsal T., Xu D.K., Lekbach Y., Gu T.Y. (2018). Microbiologically influenced corrosion and current mitigation strategies: A state of the art review. Int. Biodeterior. Biodegrad..

[2] Mansfeld F. (2007). The interaction of bacteria and metal surfaces. Electrochim. Acta.

[3] Mansfeld F., Hsu H., Örnek D., Wood T.K., Syrettc B.C. (2002). Corrosion control using regenerative biofilms on Aluminum 2024 and Brass in different media. J. Electrochem. Soc..

[4] Li S.L., Li L., Qu Q., Kang Y.X., Zhu B.L., Yu D.T., Huang R. (2019). Extracellular electron transfer of *Bacillus cereus* biofilm and its effect on the corrosion behaviour of 316L stainless steel. Colloid Surf. B.

[5] Li L., Li S.L., Qu Q., Zuo L.M., He Y., Zhu B.L., Li C. (2017). *Streptococcus Sanguis* biofilm architecture and its influence on titanium corrosion in enriched artificial saliva. Materials.

[6] Vigneron A., Head I.M., Tsesmetzis N. (2018). Damage to offshore production facilities by corrosive microbial biofilms. Appl. Microbiol. Biotechnol..

[7] San N.O., Nazır H., Dönmez G. (2014). Microbially influenced corrosion and inhibition of nickel-zinc and nickel-copper coatings by *Pseudomonas aeruginosa*. Corros. Sci..

[8] Guo Z.W., Liu T., Cheng Y.F., Guo N., Yin Y.S. (2017). Adhesion of *Bacillus subtilis* and *Pseudoalteromonas lipolytica* to steel in a seawater environment and their effects on corrosion. Colloids Surf. B.

[9] Duan J.Z., Wu S.R., Zhang X.J., Huang G.Q., Du M., Hou B.R. (2008). Corrosion of carbon steel influenced by anaerobic biofilm in natural seawater. Electrochim. Acta.

[10] Batmanghelich F., Li L., Seo Y. (2017). Influence of multispecies biofilms of *Pseudomonas aeruginosa* and *Desulfovibrio vulgaris* on the corrosion of cast iron. Corros. Sci..

[11] Örnek D., Wood T.K., Hsu C.H., Mansfeld F. (2002). Corrosion control using regenerative biofilms (CCURB) on brass in different media. Corros. Sci..

[12] Abriouel H., Franz C.M., Ben Omar N., Gálvez A. (2011). Diversity and applications of *Bacillus* bacteriocins. FEMS Microbiol. Rev..

[13] Ivanova E.P., Vysotskii M.V., Svetashev V.I., Nedashkovskaya O.I., Gorshkova N.M., Mikhailov V.V., Yumoto N., Shigeri Y., Taguchi T., Yoshikawa S. (1999). Characterization of *Bacillus strains* of marine origin. Int. Microbiol..

[14] Siefert J.L., Larios Sanz M., Nakamura L.K., Slepecky R.A., Paul J.H., Moore E.R., Fox G.E., Jurtshuk P. (2000). Phylogeny of marine *Bacillus* isolates from the Gulf of Mexico. Curr. Microbiol..

[15] Miranda C.A., Martins O.B., Clementino M.M. (2008). Specieslevel identification of *Bacillus* strains isolates from marine sediments by conventional biochemical, 16S rRNA gene sequencing and inter-tRNA gene sequence lengths analysis. Antonie Van Leeuwenhoek.

[16] Qu Q., He Y., Wang L., Xu H.T., Li L., Chen Y.J., Ding Z.T. (2015). Corrosion behavior of cold rolled steel in artificial seawater in the presence of *Bacillus subtilis* C2. Corros. Sci..

[17] Örnek D., Jayaraman A., Wood T.K., Sun Z., Hsu C.H., Mansfeld F. (2001). Pitting corrosion control using regenerative biofims on aluminium 2024 in artificial seawater. Corros. Sci..

[18] Abdoli L., Suo X.K., Li H. (2016). Distinctive colonization of *Bacillus* sp. bacteria and the influence of the bacterial biofilm on electrochemical behaviors of aluminum coatings. Colloids Surf. B.

[19] Wadood H.Z., Rajasekar A., Ting Y.P., Sabari A.N. (2015). Role of *Bacillus subtilis* and *Pseudomonas aeruginosa* on corrosion behaviour of stainless steel. Arab. J. Sci. Eng..

[20] Li Y., Liu G.W., Zhai Z.J., Liu L.N., Li H.W., Yang K., Tan L.L., Wan P., Liu X.Q., Ouyang Z.X. (2014). Antibacterial properties of magnesium in vitro and in an in vivo model of implant associated methicillin-resistant *Staphylococcus aureus* infection. Antimicrob. Antimicrob. Agents Chemother..

[21] Qin H., Zhao Y.C., An Z.Q., Cheng M.Q., Wang Q., Cheng T., Wang Q.J., Wang J.X., Jiang Y., Zhang X.L. (2015). Enhanced antibacterial properties, biocompatibility, and corrosion resistance of degradable Mg-Nd-Zn-Zr alloy. Biomaterials.

[22] Tie D., Feyerabend F., Müller W.D., Schade R., Liefeith K., Kainer K.U., Willumeit R. (2013). Antibacterial Biodegradable Mg-Ag Alloys. Eur. Cells Mater..

[23] Bbott T.B. (2015). Magnesium: Industrial and research developments over the last 15 years. Corrosion.

[24] Aghion E., Bronfin B. (2000). Magnesium alloys development towards the 21st century. Mater. Sci. Forum.

[25] Liu J.H., Song Y.W., Shan D.Y., Han E.H. (2016). Different microgalvanic corrosion behavior of cast and extruded EW75 Mg alloys. J. Electrochem. Soc..

[26] Arrabal R., Pardo A., Merino M.C., Mohedano M., Casajús P., Merino S. (2010). Al/SiC thermal spray coatings for corrosion protection of Mg-Al alloys in humid and saline environments. Surf. Coat. Technol..

[27] Zhang J., Hiromoto S., Yamazaki T., Niu J.L., Huang H., Jia G.Z., Li H.Y., Ding W.J., Yuan G.Y. (2016). Effect of macrophages on in vitro corrosion behavior of magnesium alloy. J. Biomed. Mater. Res. A.

[28] Xin Y., Hu T., Chu P.K. (2011). In vitro studies of biomedical magnesium alloys in a simulated physiological environment: A review. Acta Biomater..

[29] Hornberger H., Virtanen S., Boccaccini A.R. (2012). Biomedical coatings on magnesium alloys-A review. Acta Biomater..

[30] Zhang S.Y., Li Q., Chen B., Xu S.Q., Fan J.M., Luo F. (2010). Electrodeposition of zinc on AZ91D magnesium alloy pre-treated by stannate conversion coatings. Mater. Corros..

[31] LindstrÖm R., Johansson L.G., Svensson J.E. (2003). The influence of NaCl and CO_2_ on the atmospheric corrosion of magnesium alloy AZ91. Mater. Corros..

[32] Liao J.S., Hotta M. (2016). Corrosion products of field-exposed Mg-Al series magnesium alloys. Corros. Sci..

[33] Merino M.C., Pardo A., Arrabal R., Merino S., Casajús P., Mohedano M. (2010). Influence of chloride ion concentration and temperature on the corrosion of Mg-Al alloys in salt fog. Corros. Sci..

[34] Liu Y.H., Wang Q., Song Y.L., Zhang D.W., Yu S.R., Zhu X.Y. (2009). A study on the corrosion behavior of Ce-modified cast AZ91 magnesium alloy in the presence of sulfate-reducing bacteria. J. Alloys Compd..

[35] Zhu X.Y., Liu Y.H., Wang Q., Liu J.A. (2014). Influence of sulfate-reducing bacteria on the corrosion residual strength of an AZ91D magnesium alloy. Materials.

[36] Cui Y.Y., Li J., Ding Q.M. (2017). Research of microorganism corrosion properties of 2024-T31 Aluminum-Magnesium alloy in Oil-Water system. Int. J. Corros..

[37] Qu Q., Li S., Li L., Zuo L., Ran X., Qu Y., Zhu B. (2017). Adsorption and corrosion behaviour of *Trichoderma harzianum* for AZ31B magnesium alloy in artificial seawater. Corros. Sci..

[38] Qu Q., Wang L., Li L., He Y., Yang M., Ding Z.T. (2015). Effect of the fungus, *Aspergillus niger*, on the corrosion behaviour of AZ31B magnesium alloy in artificial seawater. Corros. Sci..

[39] Flemming H.C., Neu T.R., Wozniak D.J. (2007). The EPS matrix: The “house of biofilm cells”. J. Bacteriol..

[40] Li S.X., Bacco A.C., Birbilis N., Cong H.B. (2016). Passivation and potential fluctuation of Mg alloy AZ31B in alkaline environments. Corros. Sci..

[41] Poursaee A. (2010). Determining the appropriate scan rate to perform cyclic polarization test on the steel bars in concrete. Electrochim. Acta.

[42] Itagaki M., Suzuki T., Watanabe K. (1998). Anodic dissolution of Fe-Mo in sulfuric acid solution as investigated by electrochemical impedance spectroscopy combined with channel flow double electrode. Corros. Sci..

[43] Qu Q., Wang L., Chen Y.J., Li L., He Y., Ding Z.T. (2014). Corrosion behavior of titanium in artificial saliva by lactic acid. Materials.

[44] Liu H.W., Gu T.Y., Asif M., Zhang G.A., Liu H.F. (2017). The corrosion behavior and mechanism of carbon steel induced by extracellular polymeric substances of iron-oxidizing bacteria. Corros. Sci..

[45] Yu L., Duan J.Z., Du X.Q., Huang Y.L., Hou B.R. (2013). Accelerated anaerobic corrosion of electroactive sulfate-reducing bacteria by electrochemical impedance spectroscopy and chronoamperometry. Electrochem. Commun..

[46] Chang B.Y., Park S.M. (2010). Electrochemical impedance spectroscopy. Annu. Rev. Anal. Chem..

[47] Li Y.C., Xu D.K., Chen C.F., Li X.G., Jia R., Zhang D.W., Sande W., Wang F.H., Gu T.Y. (2018). Anaerobic microbiologically influenced corrosion mechanisms interpreted using bioenergetics and bioelectrochemistry: A review. J. Mater. Sci. Technol..

[48] Jia R., Tan J.L., Jin P., Blackwoodb D.J., Xu D.K., Gu T.Y. (2018). Effects of biogenic H_2_S on the microbiologically influenced corrosion of C1018 carbon steel by sulfate reducing *Desulfovibrio vulgaris* biofilm. Corros. Sci..

[49] Huang Y., Zhou E.Z., Jiang C.Y., Jia R., Liu S.J., Xu D.K., Gu T.Y., Wang F.H. (2018). Endogenous phenazine-1-carboxamide encoding gene *PhzH* regulated the extracellular electron transfer in biocorrosion of stainless steel by marine *Pseudomonas aeruginosa*. Electrochem. Commun..

[50] Mageshwari K., Sathyamoorthy R. (2012). Studies on photocatalytic performance of MgO nanoparticles prepared by wet chemical method. Trans. Indian Inst. Met..

[51] Moradi M., Song Z.L., Tao X. (2015). Introducing a novel bacterium *Vibrio neocaledonicus* sp., with the highest corrosion inhibition efficiency. Electrochem. Commun..

[52] Dutta A., Bhattacharyya S., Kundu A., Dutta D., Das A.K. (2016). Macroscopic amyloid fiber formation by *Staphylococcal* biofilm associated SuhB protein. Biophys. Chem..

[53] San N.O., Nazır H., Dönmez G. (2013). The effect of *Aeromonas eucrenophila* on microbiologically induced corrosion of nickel-zinc alloy. Int. Biodeter. Biodegr..

[54] Naik U.C., Srivastava S., Thakur I.S. (2012). Isolation and characterization of *Bacillus cereus* IST105 from electroplating effluent for detoxification of hexavalent chromium. Environ. Sci. Pollut. Res..

[55] Peron M., Torgersen J., Berto F. (2017). Mg and its alloys for biomedical applications: Exploring corrosion and its interplay with mechanical failure. Metals.

[56] Pestova E., Millichap J.J., Noskin G.A., Peterson L.R. (2000). Intracellular targets of moxifloxacin: A comparison with other fluoroquinolones. J. Antimicrob. Chemother..

[57] Sathyamoorthy R., Mageshwari K., Mali S.S., Priyadharshini S., Patil P.S. (2013). Effect of organic capping agent on the photocatalytic activity of MgO nanoflakes obtained by thermal decomposition route. Ceram. Int..

[58] Sunde M., Kwan A.H., Templeton M.D., Beever R.E., Mackay J.P. (2008). Structural analysis of hydrophobins. Micron.

[59] Klotz S.A., Drutz D.J., Zajic J.E. (1985). Factors governing adherence of *Candida* species to plastic surfaces. Infect. Immun..

[60] An Y.H., Friedman R.J. (1998). Concise review of mechanisms of bacterial adhesion to biomaterial surfaces. J. Biomed. Mater. Res..

