# Supplementary material for: Temporary Inhibition of the Corrosion of AZ31B Magnesium Alloy by Formation of Bacillus subtilis Biofilm in Artificial Seawater
Source: Materials (Basel). 2019 Feb 10;12(3):523. doi: 10.3390/ma12030523 (PMC6384576; doi:10.3390/ma12030523)
Supplement: Supplementary file 1 [file materials-12-00523-s001.pdf]

materials

MDPI

Article

Temporary Inhibition of the Corrosion of AZ31B Magnesium Alloy by Formation of *Bacillus Subtilis* Biofilm in Artificial Seawater

Yaxin Kang [1], Lei Li [2,*], Shunling Li [1], Xin Zhou [1], Ke Xia [1], Chang liu [1] and Qing Qu [1,*]

1 School of Chemical Science and Technology, Yunnan University, Kunming 650091, China; 12016000441@ynu.edu.cn (Y.K.); 12015001053@ynu.edu.cn (S.L.); spyxzhou@163.com (X.Z.); 18323999665@163.com (K.X.); cliu6@mail.ynu.edu.cn (C.L.)

2 State Key Laboratory for Conservation and Utilization of Bio-resources in Yunnan, Yunnan University, Kunming 650091, China;

* Correspondence: quqing@ynu.edu.cn (Q.Q.); leelei@ynu.edu.cn (L.L.)

Received: date; Accepted: date; Published: date

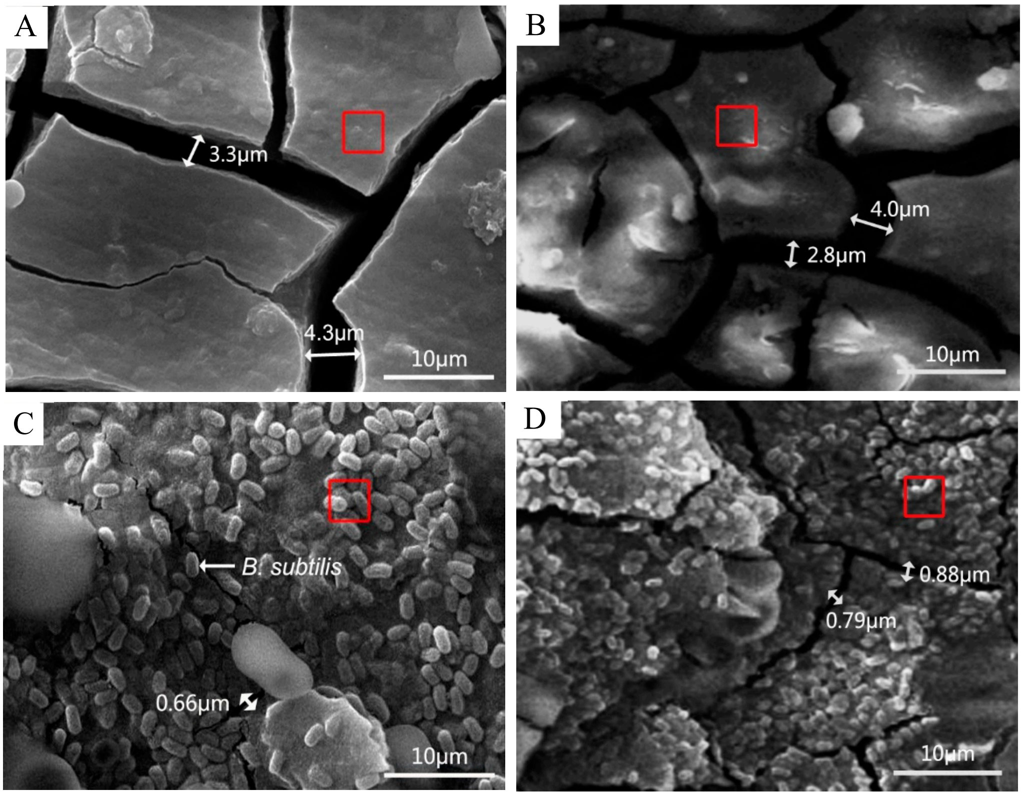

Figure S1. SEM images of AZ31B magnesium alloy specimens before or after electrochemical measurement in control group (before: A, after: B) and *B. subtilis* presence group (before: C, after: D) at 48 h. (high voltage: 20 kV).

Table S1. Elemental compositions (wt %) of the surface of AZ31B magnesium alloy specimens before or after electrochemical measurement in control group and *B. subtilis* presence group.

Element (wt %)		C	O	Na	Mg	Al	P	S	Cl	Ca	Others
The sterile control group	before	0.81	23.63	0.55	62.52	3.48	0.67	0.37	0.32	7.65	$<$0.001
	after	1.04	17.68	0.73	69.71	3.52	0.35	0.28	0.36	4.53	$<$0.001
The *B. subtilis* presence group	before	6.11	33.39	2.35	31.61	4.06	11.90	0.57	2.40	7.61	$<$0.001
	after	7.92	30.10	1.54	36.40	6.53	9.29	0.61	1.24	6.37	$<$0.001

© 2019 by the authors. Submitted for possible open access publication under the terms and conditions of the Creative Commons Attribution (CC BY) license (http://creativecommons.org/licenses/by/4.0/).
